# Lessons Learned from Co-Designing a Digital Health App for Foster Youth: Development and Usability Study

**DOI:** 10.2196/73281

**Published:** 2025-12-09

**Authors:** Johanna B Folk, Juan Carlos Gonzalez, Margareth V Del Cid, Elizabeth McBride, Tylia Lundberg, Alison Czopp, Ifunanya Ezimora, Lisa R Fortuna, Marina Tolou-Shams

**Affiliations:** 1 Department of Psychiatry and Behavioral Sciences, School of Medicine, University of California, San Francisco, 1001 Potrero Ave, San Francisco, CA, 94110, United States, 1 415-602-9521; 2 Department of Learning and Teaching, School of Leadership and Education Sciences, University of San Diego, San Diego, CA, United States; 3 San Diego State University/University of California, San Diego Joint Doctoral Program in Clinical Psychology, San Diego, CA, United States; 4 Department of Psychiatry and Neuroscience, School of Medicine, University of California, Riverside, Riverside, CA, United States

**Keywords:** foster care, adolescent, young adult, behavioral intervention technology, mHealth, co-design, app development, mental health, social determinants of health, behavioral health app

## Abstract

**Background:**

Foster youth experience high rates of unmet mental health and substance use needs, while simultaneously facing numerous barriers to accessing and engaging in community-based services. Behavioral intervention technologies (BITs) are promising for overcoming some of the barriers to service engagement, particularly when designed in collaboration with the intended users.

**Objective:**

This study describes lessons learned from a 31-month process of co-designing FostrSpace (Chorus Innovations, Inc), a BIT to address social determinants of health and behavioral health needs among foster youth. Our overall aim is to provide a roadmap for other scholars wishing to co-design BITs with minoritized youth that have the potential to address social determinants of health and increase access to and engagement in behavioral health care.

**Methods:**

The co-design process of creating FostrSpace included 5 phases: design, development, launch, testing and evaluation, and iterative refinement. We describe the activities conducted during each phase, as well as the resultant FostrSpace application. In-application FostrSpace usage data were collected as part of a quality improvement effort to iteratively refine the application; during registration, all youth signed a user agreement that detailed data usage.

**Results:**

FostrSpace usage data were collected from 40 youth (32/40, 78% aged 18‐26 years; 8/40, 20% 13‐17 years). Based on the resource needs checklist at sign-up, youth sought resources in the domains of emotional wellness (29/40; 72.5%), health care (17/40, 42.5%), housing (16/20, 40%), transportation (15/40, 37.5%), employment (15/40, 37.5%), school (13/40, 32.5%), food (12/40, 30%), family (11/40, 27.5%), and legal (7/40, 17.5%) resources, or other or not sure (16/20, 40%). Fifteen youth accessed support from the personal care navigator. Fourteen youth completed the emotional wellness questionnaire (EWQ) and identified substance use, depression, anger and irritability, mania, anxiety, somatic symptoms, and sleep problems as areas of concern. Seven of these youth initiated behavioral health services with a FostrSpace clinician.

**Conclusions:**

Engaging in participatory co-design of BITs with foster youth and other minoritized communities requires careful attention to power dynamics. Creating a space where co-designers feel there is mutual benefit to engaging in the process and it is psychologically safe to share their experiences is crucial for success. We describe lessons learned from engaging in this co-design work, including how it relates to decisions about the technology (eg, balancing youth privacy with the burden of the login process), working with third-party developers (eg, ensuring technology development partners have sufficient knowledge about the population you are co-designing with to meaningfully engage with them), and considerations for the strategic embedding of technology-based interventions within existing systems of care to promote uptake.

## Introduction

### Background

Foster youth, of whom there are over 350,000 across the United States [1], experience high rates of behavioral health (mental health and substance use) needs, yet simultaneously face numerous barriers to accessing and engaging in community-based behavioral health care [2,3]. Compared to their same-age peers, foster youth exhibit higher rates of psychiatric disorders (eg, major depressive disorder and posttraumatic stress disorder), suicidality (eg, ideation and attempt) [4], and substance use and are more likely to initiate substance use at earlier ages [5]. Data also suggest that among foster youth 11‐14 years old who had clinically elevated mental health concerns, only 26% used services [6]. These gaps in access often continue through transition age years (16‐24 years), with youth reporting ongoing barriers to care such as mistrust of the system and transportation challenges [7].

These observed inequities in behavioral health needs and services access are not a result of individual deficits, but rather, a product of complex social and systemic issues, such as poverty and structural racism, that disproportionately impact minoritized groups [8], including foster youth. Of note, families racialized as Black, Indigenous, and Latine are consistently overrepresented in the child welfare system [9,10], with Black youth in particular being more likely to be removed from their homes [11,12].

A growing body of literature has associated behavioral health needs with social determinants of health (SDoH), the social conditions into which a youth is born and grows [13]. Foster youth, who are disproportionately from minoritized ethnic and racial groups, are disadvantaged by multiple SDoH, for example, early childhood exposure to violence, child maltreatment [14], and unstable housing as a result of placement transitions. They are also less likely to access higher education and complete high school [15,16], more likely to experience economic hardship, and less likely to access needed services compared to nonfoster youth peers [6]. Thus, developing accessible supports for foster youth to address unmet behavioral health needs and reduce the undue burden of SDoH is essential. Ensuring these supports acknowledge root cause issues (eg, structural racism) is equally important to securing lasting social change [17].

Common barriers to accessing and engaging in evidence-based interventions among foster youth include frequent relocations due to multiple placement transitions, complex and multifaceted treatment needs, and services that are not designed with their unique experiences in mind [18-21]. Technology-based interventions are promising for overcoming some of these barriers, particularly when designed in collaboration with the intended users (ie, foster youth). The present study describes lessons learned from co-designing a technology-based intervention with and for foster youth to address SDoH and behavioral health needs. While terms like “users” and “end users” are commonly reported in the field to refer to those engaging in the co-design process, we chose instead to center terms such as “youth” and “foster youth” to communicate that these young people are respected and valued regardless of their relationship to the technology in development.

### Promise of Behavioral Intervention Technologies

Developing, testing, and implementing technology-based strategies to address documented barriers to care is critical in the journey toward an equitable behavioral health system where foster youth can access the support they desire and need. Technology has increasingly been leveraged to address inequities among minoritized racial and ethnic groups [22,23] and to deliver interventions to foster youth and their families [24,25]. Behavioral intervention technologies (BITs; “websites, software, mobile apps, and sensors designed to help users address or change behaviors, cognitions, and emotional states”; [26]) have the potential to support foster youth in directly accessing resources to address SDoH and behavioral health needs [27]. Mental health apps—one type of BIT—have proliferated, with estimates suggesting over 20,000 exist for download [28]. BITs for youth can promote easy access to behavioral health care by providing intervention at any time and allowing for personalization [29,30].

However, simple repackaging of evidence-based interventions for digital use may be insufficient to address existing inequities. Research has shown that despite an influx in the number of BITs developed, engagement with these technologies continues to be low [31]. Participatory methods that engage youth from the start of BIT development have been proposed as one potential solution to increase engagement [32,33]. Of the existing mental health apps for young people, however, only 30 have published descriptions of being co-designed with the youth they intend to serve, and only 3 were co-designed specifically with minoritized youth communities (eg, indigenous youth, lesbian, gay, and bisexual youth) [32]; this paucity of literature suggests significant room for the field to improve in its use of participatory co-design approaches with youth.

### Participatory Co-Design of BITs

Co-design approaches to developing BITs are intended to center youth involvement through a range of user-centered design principles, such as creative collaboration, iterative design, and acknowledgment of power differentials [32,34]. Recentering minoritized communities through such processes is also one of many necessary steps toward countering structural oppression within existing systems of care [35,36]. But the term “co-design” has been used in the literature to reference a continuum of involvement, ranging from brief consultation to more intensive, integrated involvement through mutual partnership. Greater specificity is needed to understand the utility and application of co-design approaches [32,33] and to avoid the potential of harm being perpetuated through the use of these very methods when not applied thoughtfully [37].

Participatory informatics is one approach to the co-design of BITs [38-40]. Drawing on principles of community-partnered participatory research (eg, equity and power sharing) and user-centered design (eg, active user participation in design), participatory informatics involves including people from the population you intend to serve as equitable members throughout the design process [38,39]. This approach does not require the youth to have technological expertise and instead places value on their lived expertise. Participatory informatics is therefore able to address community priorities through tools that are engaging and relevant, while also increasing co-designers’ own perceived competency in creating BITs, fostering a culture of co-leadership related to the work, and promoting sustained engagement with BITs; additional information on how we applied this to our co-design process is outlined below. Participatory informatics has been used in studies with ethnically and racially minoritized communities to address a wide range of health needs [38,41,42] and within a variety of health care settings [41,43]. To our knowledge, this approach has yet to be applied with foster youth.

### This Study

Participatory co-design of BITs with minoritized young people, such as foster youth, requires thoughtful attention to power dynamics, varying levels of technology access and literacy, and a range of SDoH needs, among other considerations. To support other scholars interested in co-designing BITs with minoritized youth to address SDoH and increase access to and engagement in behavioral health care, the current paper presents lessons learned from a 31-month participatory co-design process of a BIT (“FostrSpace”; Chorus Innovations, Inc) created by and for foster youth in California. We define the app development process (co-design and its benefits), provide a detailed description of the FostrSpace platform, and summarize lessons learned. A focus is placed on how participatory co-design was used in order to center the voices of foster youth, allowing them to speak to their needs and the impact of health inequities. For example, youth co-designers described domains of resources and needs they wanted represented within the FostrSpace app; the research team then presented the SDoH framework as a way to organize the app (ie, app features and content were organized around major areas of SDoH, including health care access and quality, educational access, and economic stability). App usage data, collected as part of quality improvement efforts, is used to understand the behavioral health and resource needs of users. This work builds upon our prior examinations of co-designers’ reflections on the participatory co-design process [44] and initial usage data for FostrSpace in an ongoing hybrid implementation-effectiveness trial in partnership with Court Appointed Special Advocate (CASA) programs [45]. Through this description of our methodological application of co-design with foster youth, we aim to provide relevant insights for other scholars and ultimately to encourage the use of such methods to advance behavioral health equity for minoritized youth.

## Methods

### Recruitment

### Co-Design Participants and Process

Co-design of FostrSpace began in the planning stages. Idea-sharing workgroups, advertised using digital recruitment flyers distributed by Bay Area organizations serving foster youth, were held with 5 present and former foster youth (ages 16‐26 years); two 90-minute groups were held, one with 3 participants and the other with 2. Workgroup participants were compensated with a US $75 gift card and invited to join the FostrSpace Advisory Board (FAB). FAB members (4 of the 5 workgroup members) were hired as university employees and paid an hourly rate (based on their individual compensation package, which was determined by education level and experience as set by the university) for participation in the co-design process. The FAB consisted of 4 transition-age youth; their personal background and experience co-designing the initial version of FostrSpace have been described elsewhere [44]. For 18 months, the FAB participated in weekly 90‐ to 120-minute meetings with a team of academic clinical researchers (academic team) and developers at Chorus Innovations, Inc, a Health Insurance Portability and Accountability Act (HIPAA)–compliant web-based app development platform.

**Figure 1.**
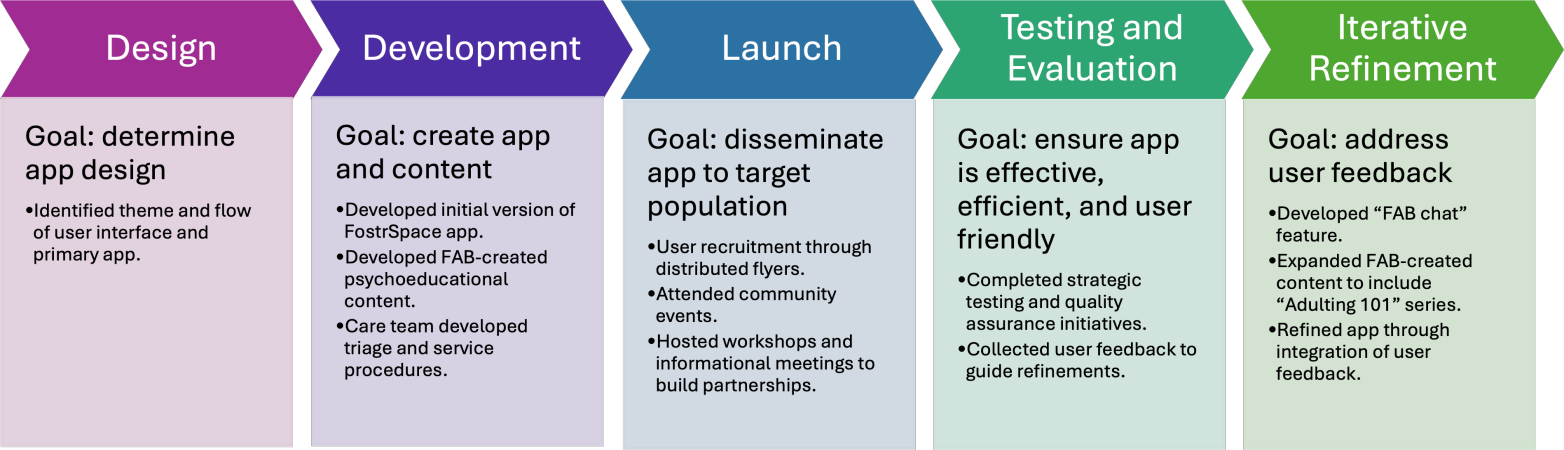
FostrSpace app co-design process.

The process of creating FostrSpace included 5 phases (see Figure 1): design, development, launch, testing and evaluation, and iterative refinement. In the design phase, meetings focused on gathering feedback from the FAB about the app interface, framework, features, and functionality. During these meetings, the FAB team created the app name and theme and identified the primary features of the app. These app features organically fit within an SDoH framework, which addresses key areas of social impact that influence youth behavioral health outcomes (eg, health care access and quality, education access and quality, social and community context, economic stability, and neighborhood and built environment). Co-designers engaged in shared decision-making about FostrSpace features and design, prioritizing the FAB preferences at all stages and taking into account what was possible to create within the existing platform and resources. In the development phase, the Chorus team built the initial version of FostrSpace, the FAB created psychoeducational and testimonial app content, and the care team (ie, behavioral health providers and personal care navigators hired to provide in-app services) developed clinical workflows and triage processes. All content was tested extensively by the FAB and academic team and iteratively refined to improve usability. The launch phase focused on dissemination and outreach to enroll youth to test the app. In the testing and evaluation phase, the team engaged in ongoing strategic testing and quality assurance initiatives to ensure the app was effective, efficient, and user-friendly. Qualitative feedback from youth, including the FAB, informed changes to the app in the iterative refinement phase. The “FAB Chat,” which offers daily in-app peer support from our FAB team to cultivate community, was added during this phase in response to youth feedback. Youth also requested content focused on SDoH relevant to the transition to adulthood, which inspired the FAB to create the “Adulting 101” content and video series, including independent living resources (eg, how to apply to a job or food benefits). Finally, also during the iterative refinement phase, the FostrSpace app and academic team were awarded a federal Children and Youth Resilience Challenge grant, which spanned from September 2023 to May 2024. Through this challenge, our team added 2 new FAB members with lived experience in the foster care system (2 existing members became inactive at this time due to competing personal demands) and added a measure of youth resilience into the FostrSpace app. The FAB worked, on average, 4 hours each week during this time to create new app content, test and refine existing content and features, and engage in outreach activities.

FAB members had a range of experiences in both the traditional foster care system and kinship/relative care and represented a diverse set of backgrounds across race, ethnicity, sexual orientation, gender, disability status, and socioeconomic status (eg, White, White-passing, mixed Asian-Hispanic, Mexican-Latino, and Chicana; nonbinary, woman, and men).

### Co-Designed Product: the FostrSpace App

Combining “Foster” and “‘Space,” the name FostrSpace was selected in partnership with the FAB to easily identify the app as a welcoming space for foster youth. The app uses imagery such as astronauts, planets, stars, and rocket fuel to create a lively and developmentally appropriate virtual space for users to explore their mental wellness.

**Figure 2.**
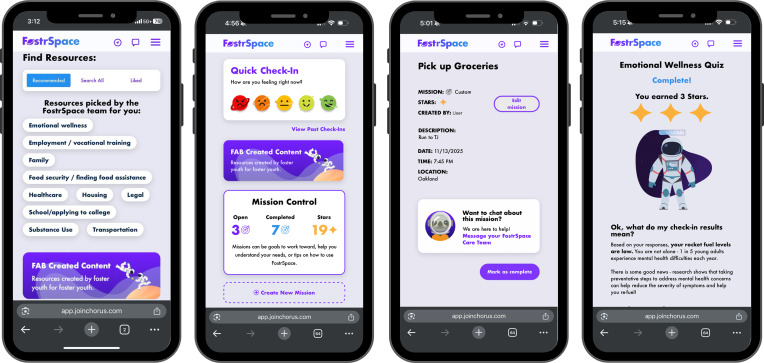
FostrSpace app screenshots.

FostrSpace provides access to a wide range of both self-guided and service-oriented features (eg, navigation and clinical services). Self-guided features include psychoeducational content (eg, breathing exercises and regulation skills) and a curated resource directory organized by SDoH area (eg, school and applying to college, employment and training, health care; see Figure 2, Find Resources screenshot) across several SDoH domains (eg, educational, occupational, health care, and legal). Self-guided assessments for emotional wellness and resource needs are incorporated to personalize app content for foster youth and inform the care team of the youth’s self-identified priority needs. Synchronous service-oriented features include chat-based support from peers with lived experience in the foster care system, resource linkage support from a personal care navigator, and behavioral health services via telehealth, which are available weekdays except on holidays from 9 AM to 6 PM (see Table 1).

Foster youth can engage with the app independently or with support from their care team, with features designed to build self-awareness (eg, mood tracking) and promote autonomy (eg, missions). For example, youth have the option to complete a single-item mood check-in question upon each login on the landing page of the app (see Figure 2, Quick Check-In screenshot), with the option to briefly describe their mood in a write-in section; responses are logged over time so youth can revisit them for personal tracking. Youth can also set and track goals (eg, completion of app tutorials and submission of job applications) through “Missions” (see Figure 2, Pick Up Groceries screenshot). When missions are self-generated, foster youth are prompted to add a description of their goal, a timeline on intended goal completion, and log any relevant hyperlinks they might need to complete the task (eg, job application site in the case of a vocational goal). If youth engage in services, goals can also be assigned by the care team to facilitate completion of app-based content, therapy goals, or in-app assessments. Peer support with mood tracking and goal organization is accessible through in-app chat with the FAB.

Youth seeking emotional wellness resources are invited to complete the in-app Emotional Wellness Questionnaire (EWQ; a co-designed behavioral health assessment including items from the *DSM-5* [*Diagnostic and Statistical Manual of Disorders* {Fifth Edition}], Self-Rated Level 1 Cross-Cutting Symptom for adults [18+ years] and youth [11-17 years] measuring frequency and severity of past 2-week symptoms [41]). All respondents receive feedback on their responses (see Figure 2, Emotional Wellness Quiz screenshot with astronaut and rocket fuel metaphor for emotional wellness, ie, behavioral health intervention need) and tailored resources (eg, self-guided psychoeducational content to learn coping skills), and for those whose responses are elevated, the suggestion to connect with a FostrSpace clinician. FostrSpace navigators send an in-app chat message within 48 business hours to youth with elevated scores to offer support with connecting to a FostrSpace clinician. Foster youth may also opt to message the navigator directly to request help initiating clinical services. Navigators serve as the primary point of contact, supporting youth in accessing appropriate mental health resources. For youth not yet ready to engage with a mental health clinician (whether through FostrSpace or external services), navigators address barriers to care and co-develop a gradual, youth-paced plan to facilitate connection to desired resources. Foster youth who engage with FostrSpace clinicians are asked to complete follow-up questionnaires (ie, DSM Level 2 Cross-Cutting Symptom Measures; [46]) to inform clinical intervention needs in particular area(s) (eg, depression, anxiety, substance use). All FostrSpace clinicians are trained and experienced in providing care to youth with current or prior history of foster care system contact.

**Table 1.** FostrSpace app features and description of co-design process.

Type and feature		Description	Co-design process
Self-guided and asynchronous			
Resource directory		A curated directory where youth can independently find local resources and services, such as food pantries, job programs, and free clinics.	The FAB^a^ identified key resource categories based on their lived experience and recommended local, county-specific resources.
Peer- created resources		The FAB created videos, handouts, and guides to help youth navigate adulthood and build independence, including FAB-led skill-building videos to promote healthy coping.	The FAB developed coping strategy videos and collaborated with the clinical team on script development. FAB members prioritized and led resource creation based on independent living needs in addition to coping skills.
Assessments		Youth can access assessments like the Emotional Wellness Questionnaire to explore their behavioral health or the Resource Needs Survey to identify resources. Youth also complete a resource needs checklist at sign-up to indicate sought-out resources.	The FAB advocated for an in-app emotional wellness check-in and refined screening tool questions to remove stigmatizing language.
Mood tracking		The FostrSpace Mood Tracker allows for daily mood tracking, which provides opportunities for self-awareness and empowers youth to take control of their emotional states.	The FAB proposed a simple emoji-based mood tracker, with the option to add written descriptions. The design team provided emoji options, and the FAB selected the ones used in the app.
Missions		Missions in the app help organize goals. For example, completing specific tasks, such as attending health appointments.	The FAB emphasized the importance of goal setting and tracking in the app, providing design suggestions to match its space theme.
Service and synchronous			
Peer support		The FAB provides daily in-app support via the “FAB Chat,” helping youth access resources and receive peer guidance.	The FAB highlighted the importance of built-in peer support to foster community. The research team structured workflows for the FAB chat function.
Navigation services		Personal care navigators help youth access social services, health care, and other resources to meet their basic needs (eg, supporting with building skills to complete paperwork, schedule appointments, etc).	The research team shared successful navigation models, while FAB emphasized the importance of opt-in services to prioritize foster youth autonomy.
Clinical services		Licensed clinicians provide evidence-based behavioral health services via telehealth.	The FAB communicated the importance of opt-in clinical services to prioritize foster youth autonomy, while the research team structured workflows for access.
In-app chat		The in-app chat connects youth with their care team, including the FAB.	The FAB advocated for in-app chat to reduce barriers to support by enabling real-time, confidential communication without phone calls or emails. Developers supported the chat feature build-out, allowing users to ask questions, share updates, and seek guidance while fostering connectivity and reducing isolation. The research team provided feedback on its functionality through testing.

a FAB: FostrSpace Advisory Board.

### Launch Phase: Enrollment and Outreach

Outreach efforts included contacting 220 individuals across 153 schools through the Guardian Scholars Program (designed to support foster youth in higher education), 1805 school personnel across 58 counties through the California Department of Education Services, and 42 community-based organizations; in total, we outreached to 2300 contacts (from September 1, 2021, to April 13, 2024). In addition to cold emails and direct contact with community organizations that serve foster youth, our team presented at 5 state-level child welfare leadership meetings and hosted 8 virtual events for the statewide foster youth community directly, where training in policy and advocacy was provided and access to the app was highlighted. As described below, despite this extensive outreach, enrollment during the evaluation period was lower than anticipated (n=40).

### Testing and Evaluation

Data reported in the current manuscript were collected between April 25, 2022, and November 14, 2024 in-app and used solely for quality improvement purposes to improve FostrSpace over time. An in-app data use agreement was signed by each youth as part of the FostrSpace sign-up process. Youth were provided with the following statement: “The data collected will be used to maintain the FostrSpace app’s functionality and performance and support FostrSpace service provisions. Information collected through FostrSpace could include your name, contact information, responses to surveys, and/or engagement activity (log-ins, completion of surveys). Information about who has access to your data and where it is stored can be accessed within the app under the “My Data” section. At any time, you can request to be removed from the platform and have your data deleted.” All data were stored within the FostrSpace app on the Chorus platform and used only for internal quality improvement purposes. Data sent to or from the FostrSpace app is transmitted over encrypted, industry-standard connections like SSL/TLS. The server is protected using standard practices, including being located behind a network firewall and accessible only by designated users. No data are stored locally on users’ devices (such as local browser storage or temporary storage) that access FostrSpace. App usage data were analyzed using descriptive statistics to understand the behavioral health and resource needs of users.

### Ethical Considerations

The institutional review board of the University of California, San Francisco, determined that this research is exempt from institutional review board oversight according to the federal regulations summarized in Title 45 of the Code of Federal Regulations, Part 46.102(l). Demographic information was intentionally not collected from app users except for age, which was needed to determine eligibility. This decision was driven by our desire to ensure the app remained accessible and safe for a population of young people who are highly surveilled. Demographic information was collected for those engaging in services; however, given the small sample size, we do not include this information here.

## Results

App platform data were collected from 40 foster youth (32/40, 80% aged 18‐26 years; 8/40, 20% aged 13‐17 years). On average, users logged into the app 3.88 (SD 5.26; range 1‐22) times on 2.93 (SD 4.15; range 1‐16) days. Twenty-eight users (28/40, 70%) completed at least one mood check-in, and among those who did, users logged their mood an average of 3.75 (SD 4.72; range 1‐17) times in total. Twenty users (20/40, 50%) sent at least one message using the in-app chat.

Based on the resource needs checklist at sign-up, youth sought resources in the domains of emotional wellness (29/40; 72.5%), health care (17/40, 42.5%), housing (16/20, 40%), transportation (15/40, 37.5%), employment (15/40, 37.5%), school (13/40, 32.5%), food (12/40, 30%), family (11/40, 27.5%), and legal (7/40, 17.5%) resources, or other/not sure (16/40, 40%). Seventeen users accessed the resource directory, ten accessed the “Get Help Now” page with crisis resources, and 15 accessed support from the personal care navigator.

Fourteen youth completed the EWQ and identified needs related to substance use, depression, anger or irritability, mania, anxiety, somatic symptoms, and sleep problems. The most commonly endorsed concerns (scores 2+) among youth aged 13‐17 years were depressive symptoms (3/8, 37.5%) and attention difficulties (3/8, 37.5%), followed by anger (2/8, 25%), anxiety (2/8, 25%), somatic complaints (2/8, 25%), sleep disturbances (2/8, 25%), and irritability (2/8, 25%). Among youth aged 18 years and older, the most frequently reported concerns (scores 2+) were impulsivity or increased energy (Mania scale, 10/32, 31.3%), anxiety (9/32, 28.1%), depressive symptoms (9/32, 28.1%), and somatic complaints (9/32, 28.1%). Youth who score a 2+ (or 1+ on SU) on the EWQ are contacted by the FostrSpace navigator to link to a FostrSpace clinician for intervention. Of the 14 youth who scored 2+ (or 1+ on substance use) on the EWQ, seven initiated services with a FostrSpace clinician; primary symptoms included substance use, anxiety, depression, anger or irritability, and sleep disturbance, and all endorsed recent substance use, specifically cannabis use (one endorsed alcohol and cannabis use).

## Discussion

### Principal Findings: Lessons Learned and Recommendations

#### Co-Designing BITs With Foster Youth

Engaging in participatory co-design of BITs with foster youth and other minoritized communities requires careful attention to creating a space where co-designers feel there is mutual benefit to engaging in the process, are psychologically safe to share their experiences, and that power dynamics are acknowledged and attended to. This type of participatory process takes time (ie, for our team, it took 18 months of weekly co-design meetings to develop the initial version of FostrSpace), which in academic settings can feel scarce as we push against deadlines and expectations set by institutions and funders [47]. Our team was fortunate to have a flexible funding source for this project, which afforded critically needed flexibility and time to complete project goals. Regardless of funding structure, it is important for academics engaging in this type of work not to rush through it, as this can risk recreating harms caused to foster youth (eg, paternalistic decision-making). Slowing down in the research process can reduce possible harms associated with asking youth to prioritize this over more pressing needs, for example, employment or educational responsibilities for financial security and education, and may require adapting timelines to meet youth where they are. Active reflection during and after the co-design process, by all members, can help identify areas for process improvement. Duoethnography—a qualitative research methodology in which two researchers engage in a dialogic, collaborative process to explore a topic through their personal experiences and perspectives—is one approach to such reflection [33,44]. Using a duoethnographic reflection process, the FAB and academic team who co-designed the initial version of FostrSpace developed a series of recommendations, which have been detailed elsewhere [44] for creative ways to share power and build community (eg, flattening power hierarchies and building community through icebreakers) and ensure mutual benefit (eg, providing professional development opportunities such as co-authoring manuscripts and delivering presentations) among co-designers. These strategies can be used to avoid replicating problematic patterns of researchers having relationships with minoritized communities that are based primarily in extraction.

In addition, when working with young people longitudinally, it is important to recognize that developmentally appropriate transitions (eg, changes in employment) and priorities (eg, juggling competing demands with school and family) may impact sustained engagement. Our team provided flexibility throughout the co-design process whenever possible, including through meeting remotely, rescheduling meetings as co-designers’ schedules changed (including meeting in the evenings after school and work hours), and respecting natural transitions away from participation while adding new members to sustain the FAB. Such flexibility is particularly important when working with foster youth, as they may experience frequent placement changes and other forms of instability, which could impact participation.

Central to the co-creation of FostrSpace was foster youth raising in-app privacy and safety concerns. This necessitated co-designer discussions about how to practically protect youth privacy (eg, having a secure login requiring a unique username and password) and how to ensure foster youth actually understood their information was secure. The importance of transparency regarding how data are used was highlighted by the FAB, as co-designers agreed we all often click through long, technical app use agreements without truly reading the terms and conditions. In addition to a user agreement everyone sees during the initial registration process, the FAB created a “My Data” page in the app explaining who has access to the young person’s data and for what purposes, how data are stored, and who to contact with questions. Information about who has access to data and why is also presented on specific pages within the app. For example, at the beginning of the EWQ, there is a statement, “Our trained FostrSpace Care Team will have access to your results to better understand your needs.” To further promote autonomy, youth also have the option to skip questions on assessments like the EWQ, not engage with any app features they choose, and delete their FostrSpace account at any time. Without a true participatory co-design process, it is unlikely that an academic team or tech developer would have understood privacy and safety concerns in the same way that youth presented, and, for example, a “My Data” page would not likely have been a part of the app.

#### Technology Pitfalls: Addressing Barriers to BIT Engagement

In our efforts to create an app where foster youth privacy is protected, youth are required to create a unique username and password. This created an unanticipated barrier related to account verification. Specifically, the platform required youth to enter a personal email address upon registration, to which they received a confirmation link to complete registration. Several youth reported significant issues with the registration process throughout the various stages of testing and implementation of the app. While creating a password through a verification email is important for the purposes of maintaining data security in a platform containing sensitive information, this minor step in the registration process may be leading to a lack of completion of the registration process for foster youth. It is possible that different forms of account confirmation (eg, text confirmation code) could result in improved retention, which would be consistent with literature surrounding technology preferences among youth [48].

In addition to considering the technology usage patterns of the intended youth population when designing onboarding and app access workflows, we recommend seeking feedback to iteratively refine such processes, as they have the potential to hinder youth from accessing the BIT. Working closely with youth, developers, and technology partners to ensure efficient and effective registration workflows, and to iteratively refine them as needed, is critical to retaining youth in the digital space. Whereas an in-person clinic may have a welcoming administrative staff, peer support workers to help youth complete registration paperwork, or a supportive therapist explaining the pathways to accessing continued support, BITs are restricted to technical workflows where failsafe engagement is difficult to ensure. As the sophistication of artificial intelligence tools increases, teams may consider how these specific technologies could serve as digital assistants throughout the registration process, including by way of registration reminders for those who do not complete the entire flow [49].

#### Working With Third-Party Developers

The empirical literature on technology co-design offers some guidance in terms of how to consider the youth in the design process, though it is limited in the extent to which user-centered design methods are described in a way that is replicable. Some of our team members had received basic training in user-centered design methods; however, our collective BIT co-design experience was limited. Formal training in user-centered design methods may increase the extent to which research teams are able to design easy-to-use and effective interventions. Partnering with third-party developers can also be beneficial for transforming the co-design team’s vision into a functional and engaging app experience. We elected to partner with a third-party developer (Chorus Innovations, Inc) whose staff included user experience researchers and designers with formal training in this domain. Our co-design process actively involved a user experience researcher who received graduate training in user experience design and helped us to develop and facilitate user-centered design activities during the co-design meetings with the FAB. This partnership ultimately allowed us to create an app that attended to accessibility, functionality, and design.

Throughout this partnership, our team of foster youth, researchers, and developers collectively faced a number of growth areas. First, we had to develop a memorandum of understanding and scope of work to ensure the roles of the development team (ie, Chorus Innovations, Inc) were clearly outlined, including deliverables and invoicing expectations. Second, once beginning the work, we had to work together to develop a shared language; for example, what is a technical bug (eg, something not operating as it is intended to) versus a desired change (eg, something is operating as it is intended to but is not how we would like it to operate). Third, reconciling project management systems and preferences across our team and the developer was essential for tracking and communicating about issues within the app, new feature and content requests, and ensuring shared understanding of the expected timeframes for addressing milestones and unanticipated issues. We ultimately found that holding regular synchronous working meetings (at minimum weekly during the active design phase), having asynchronous communication primarily through Slack (Slack Technologies, LLC), and having shared files (through Google Drive) were necessary for communication, documenting progress on action steps, and collaborative problem-solving. At least one representative from the research and development team was present in co-design meetings with the FAB to ensure each team’s expertise was represented and factored in when making decisions. Fourth, developers may not have experience working with the specific youth population, and when co-designing with foster youth and other minoritized communities, it is essential all parties understand basic ethical considerations. Researchers may consider facilitating basic training for developer partners regarding the needs of the population being served and ethical considerations for the co-design process prior to their interaction with the intended youth population. In our process, the academic and developer teams collectively reviewed plans for co-design meetings to ensure attention both to user-centered design methods and to the FAB’s needs and psychological safety. While these plans will likely be iterative, we recommend that teams take time before beginning a co-design process to discuss these foundational items. When possible, maintaining a curious stance when approaching points of divergence can help identify when differing professional backgrounds and conventions may be the root of misunderstandings or inefficiencies. When appropriate, these trainings and discussions should be held with youth co-designers (eg, foster youth) to promote the establishment of a shared language based in cultural exchange and ensure that each member understands their roles, expectations, and communication.

#### If You Build It, Will They Come?

One of the main takeaways from the participatory co-design of FostrSpace was that simply co-designing and co-building the BIT was insufficient to drive youth engagement. Despite co-designing with youth who have lived experience in the foster care system, reaching out to 2300 contacts who directly serve foster youth, attending community events to present about Fostrspace (eg, college resource fairs), and posting flyers in public locations (eg, coffee shops or bus stops) to advertise FostrSpace, the total number of youth who have completed registration remains under 50 over a 31-month period of time. This indicates we have reached a limited portion of the roughly 19,000 eligible youth in the foster care system across the state of California [50]. This low uptake suggests a strategic, systems-embedded approach may prove more fruitful [51].

Embedding BITs within existing systems serving foster youth may increase the uptake and sustainability of BITs, particularly when using implementation science frameworks and integrating community engagement to guide dissemination and implementation [52,53]. Similar to other models of addressing service gaps, BITs can be embedded in existing systems to meet specific goals or gaps (eg, providing interim support for youth who are on long waitlists [54]), offered as adjunctive services, or advertised through sustained partnerships with systems serving foster youth [55,56]. Of note, our team is currently evaluating the effectiveness of working with CASA programs to increase FostrSpace usage by foster youth, aged 13-20 years [45]. CASAs are adult volunteers who work with youth involved in the child welfare system, typically one-to-one, in an ongoing, in-person, and long-term (average 18 months) capacity, to provide support and advocate for youth’s access to necessary resources. Using approaches within specific systems or organizations may not reach youth who are not already connected to a serving or support system; thus, an engagement and outreach approach that applies multiple strategies across multiple systems and programs that serve foster youth may maximize reach and uptake.

In addition, despite our team having interdisciplinary experiences and training, no members had a formal background in marketing, and our advertising budget was limited. Our outreach efforts relied upon direct communication with foster youth and virtual and in-person events and professionals disseminating digital flyers to potentially eligible foster youth; we were not able to track whether our flyers were disseminated or to how many young people, and it is possible some contacts ignored our outreach attempts. Using other methods of advertising, such as social media, may be promising for reaching foster youth [57]. Researchers should also consider partnering with marketing experts or seeking training in this area themselves and budgeting to allow for wide-reaching advertising that does not predominately rely on middle-person approaches to reach youth.

### Limitations

The current manuscript describes lessons learned from the process of creating FostrSpace, a BIT designed for and by foster youth. Considerations for interpreting our recommendations include that the work was conducted in a single United States state with a small FAB, which could impact generalizability; we used a web-based app platform, and challenges and opportunities may differ for other platforms such as native apps; and we did not conduct a formal evaluation of the app during the initial testing stages, but rather report available quality improvement data.

### Conclusions

Lessons learned from the process of creating FostrSpace can inform researchers seeking to co-design BITs with foster youth, as well as young people more broadly. There is no one-size-fits-all approach to engaging in co-design, and as such, we encourage researchers to provide greater specificity regarding the co-design methods used so we can advance our scientific understanding of how best to engage in this type of work. Relevant across co-design methodologies, our experience highlights the importance of active reflection throughout the process to ensure the creation of a psychologically safe and constructive co-design space where young people, academics, and developers can work together to create accessible and impactful BITs. One element of this is ensuring adequate resource investment before beginning the process (eg, available compensation for co-designers and time commitment of all involved) to avoid replicating harmful research practices of extracting from minoritized communities, as co-designing BITs can take significant time and resources. This time-intensive participatory approach can run counter to deadlines and expectations set by institutions and funders [47], and broader advocacy for the centrality of these methods to advancing health equity is needed to ensure their feasibility within existing structures.

## Abbreviations

BIT: Behavioral intervention technology
CASA: Court Appointed Special Advocate
*DSM-5*: *Diagnostic and Statistical Manual of Disorders, Fifth Edition*
EWQ: Emotional wellness questionnaire
FAB: FostrSpace Advisory Board
HIPAA: Health Insurance Portability and Accountability Act
SDoH: Social determinants of health
